# A combined behavioral and EEG investigation of facial emotion recognition in sexual offenders against minors

**DOI:** 10.1016/j.ijchp.2025.100641

**Published:** 2025-10-15

**Authors:** William Vallet, Sabine Mouchet, Mathias Poitau, Chloé Laval, Benoit Bediou, Guillaume Giret, Jerôme Brunelin

**Affiliations:** a Le Vinatier Psychiatrie Universitaire Lyon Métropole F-69500 Bron, France; b Université Claude Bernard Lyon 1, Centre National de la Recherche Scientifique, Institut National de la Santé et de la Recherche Médicale, Centre de Recherche en Neurosciences de Lyon U1028 UMR5292, PSYR2 F-69500, Bron, France; c CRIAVS Auvergne-Rhône-Alpes - Délégation de Lyon F-69500 Bron, France; d Université de Genève, Faculté de Psychologie et Sciences de l’Education, Campus Biotec, 9 Chemin des Mines 1211 Genève, Switzerland; e Digital Wellness Lab, Boston Children’s Hospital, Harvard Medical School, Boston, USA

**Keywords:** Emotion, Aggressivity, Impulsivity, Pedophilia, Child molester

## Abstract

This study addresses a significant gap in literature by investigating emotional processing in sexual offenders against minors (SOm), using a combined electroencephalographic (EEG) and behavioral approach. In the first experiment, explicit behavioral abilities related to emotional recognition and the evaluation of perceived facial emotions were assessed in 15 SOm individuals, compared to 15 matched controls. In the second experiment, participants were asked to complete an implicit emotion task involving the presentation of emotional facial expressions while EEG activity was recorded. Behavioral results revealed a significant deficit in the recognition of fearful emotions, and an altered evaluation of the valence of disgusted emotional faces, in the SOm group compared to the control group. The implicit EEG results indicated a significantly lower amplitude in the P2-LPP temporal windows across temporo-parieto-occipital locations and modulated by the emotion and age of the presented faces. Taken together, these results highlight an emotional deficit in the SOm group, further reinforcing the hypothesis that this population has specific difficulty recognizing and interpreting emotional facial expressions, particularly those conveying negative emotions. These results represent a preliminary step toward a more comprehensive understanding of the cerebral underpinnings of emotional deficits in individuals who have committed sexual offenses against minors.

## Introduction

The prevalence of sexual violence and aggression against minors constitutes a global public health and societal concern. While legal definitions of sexual violence against minors vary slightly, the term is generally understood to refer to aggression involving victims under the age of 14 to 18, depending on national legal thresholds for the age of sexual consent. The global prevalence of sexual abuse indicates that 18.9 % of women and 14.8 % of men have reported a history of abuse before the age of 18 (Cagney et al., 2025). The consequences for victims are severe and long-lasting (Banvard-Fox et al., 2020). Moreover, aggressors present a recidivism risk estimated ranging between at 10 % to 50 % for committing further sexual assaults against children (Hall & Hall, 2007). These outcomes result in a significant burden for society and highlight the need for further research to better understand the cognitive and neural underpinnings of violent behavior toward others, particularly in individuals who have committed sexual offenses against minors (SOm), as well as to improve prevention strategies and provide appropriate support. However, gaining a clearer understanding of the role of cognitive and neural processes in the etiology and maintenance of sexual offending against minors remains a complex and challenging area of investigation. One neuroscience-based approach to investigating correlates of sexual violence against minors is to examine the cognitive and neurophysiological processes underlying social cognition in SOm (Cartwright et al., 2022; Gillespie et al., 2021; Greenfield et al., 2023; Schuler et al., 2022). Social cognition refers to the cognitive processes that underline social interactions, enabling individuals to respond appropriately to the intentions, emotions, and behaviors of others. These processes which include emotion recognition, empathy, and theory of mind are essential for effective communication and social integration within a group (Adolphs, 2009; Frith, 2008). Some studies have investigated emotion recognition in SOm using explicit emotion recognition tasks compared to controls, but the results have been inconsistent. While several studies suggest potential impairment in emotion recognition among SOm, particularly in recognizing faces that signal fear, disgust, and sadness (Francis & Wolfe, 2008; Gery et al., 2009; Gillespie et al., 2015; Hudson et al., 1993; Seidel et al., 2013; Suchy et al., 2009) other studies have reported no significant deficits in emotion recognition in SOm (Gillespie et al., 2021; Oliver et al., 2009; Rodriguez & Ellis, 2018; Wegrzyn et al., 2017). Similarly, systematic reviews have highlighted the complexity and heterogeneity of the relationship between sexual offending and deficits in emotion recognition (Chapman et al., 2018; Virolle et al., 2024), emphasizing the need for further research on this topic in SOm. In addition to the limited available data, the question of how SOm responds to stimuli depicting children compared to adults has been scarcely explored at the behavioral level.

Investigating social cognition using explicit emotion recognition tasks may present limitations in this population. This issue can be addressed by using EEG, which allows for the examination of very early stages of cognitive and attentional processing. However, to the best of our knowledge, over the last decade, only two studies have specifically explored EEG activity in SOm (Knott et al., 2016; Rosburg et al., 2018) and one study has investigated juvenile individuals reporting a sexual preference for prepubescent children (Speer et al., 2022). In the first study Knott and collaborators investigated attention towards adult erotic stimuli in SOm. They reported a reduced P2 response after the stimulus onset, suggesting a possible diminished sexual interest for adults among SOm compared to controls. The second study explored the error monitoring in SOm. The authors primarily reported abnormalities in components associated with error awareness, particularly a reduced amplitude of the error positivity component (PE) (Rosburg et al., 2018). This reduced PE amplitude has also been observed in individuals with psychopathic traits (Vallet et al., 2021) and more broadly in individuals with externalizing disorders (Lutz et al., 2021) .Finally, a more recent study conducted by Speer and collaborators (Speer et al., 2022) explored the processing of emotional child and adult faces in a juvenile sample reporting a sexual preference for prepubescent children. The results showed that, regardless of emotional content, juveniles reporting a sexual preference for children exhibited significantly greater frontal negativity in response to child faces compared to adult faces.

In light of current knowledge, it appears crucial to pursue integrated investigations combining electrophysiological and behavioral measures to better characterize the cognitive functioning of individuals who have committed sexual offenses against minors. The main objective of the present study is precisely to examine potential abnormalities in emotional processing among SOm by assessing the causal relationships between behavioral and neural responses to emotional facial expressions, in comparison with a matched control group. Specifically, this study aims to explore whether atypical patterns of electrophysiological and behavior coupling emerge during the perception of emotional faces, which could provide new insights into the cognitive and neural mechanisms underlying social–emotional processing in this population.

In the current study, we aimed to examine both the behavioral and neurophysiological aspects of emotional processing in response to adult and child faces in SOm compared to matched controls. At the behavioral level, we hypothesized that SOm would exhibit reduced facial emotion recognition accuracy compared to the control group, particularly for negative emotions such as disgust and anger. We also expected that SOm might display altered intensity ratings, potentially underestimating the emotional salience of negative expressions. Furthermore, we hypothesized that these effects would be amplified when the emotional expressions were presented on child faces. Regarding neurophysiological processing, we hypothesized a significant interaction between Group and Emotion and between Group and Age, with altered EEG responses in SOm, particularly in response to child faces and negative emotions. Based on existing evidence of emotional recognition deficits in offender populations (Virolle et al., 2024), we expected to observe alterations in early and late ERP components associated with attentional and emotional processing.

## Method and materials

### Sample

In the current study, we chose to recruit all types of SOm, regardless of whether they met diagnostic criteria for pedophilia. The SOm group consisted of individuals under the age of 55, all right-handed and had been convicted of sexual offenses against minors. The inclusion criteria also required that SOm participants had no psychiatric or neurological comorbidities and were not undergoing long-term psychotropic treatment. The study was conducted on a sample of 30 right-handed male adults, including 15 individuals convicted of sexual offenses against minors and 15 controls, between July 12, 2022 and May 15, 2025. The SOm group was recruited from a forensic psychiatric facility located within the Le Vinatier, Psychiatric Hospital, which specializes in the treatment and follow-up of individuals who have committed sexual assaults (CRIAVS). The study was approved by the local ethics committee (Comité de protection des personnes, IdF, Paris, France on March 2, 2022) and by the French National Agency for the Safety of Medicines and Health Products (ANSM; 2021-A02763–38). The study was preregistered in a public database (clinicaltrials.gov identifier: NCT05383235).

In the present sample, 14 SOm were identified as having committed sexual abuse against minor under the age of 15, and 1 individual was identified as having committed sexual abuse involving a 17-year-old female. Most of the SOm offended against female victims (11 females, aged 5 to 17 (Mean = 11.86; Standard Deviation = 3.83) and 4 males victims ranged in age from 8 to 15 years (*M* = 10.67; SD = 2.42)). Regarding the relationship with the victim, 7 SOm committed offenses within an intra-familial context, 5 within an extra-familial context, and 2 in both intra- and extra-familial contexts. Four subjects were first time offenders, and 11 subjects were repeat offenders. Among the SOm sample, 7 individuals were diagnosed with pedophilia. The control participants had no personal or first-degree family history of neurological or psychiatric disorders, nor were they taking any medication. Information regarding the medication use in the SOm and control groups is reported in Table 1.

**Table 1:** Demographic sample characteristics.

	SOm	Control
n	15	15
Employment status (employed / unemployed)	14/1	14/1
Age years, mean ± SD (range)*	47.13 ± 7.1 (33–57)	37.6 ± 8.05 (28–53)
Education (years, mean ± SD)	12.0 ± 3.16	14.8 ± 3.36

SOm; sexual offenders against minors. SD; standard deviation.

⁎ no difference between group regarding age (t(28) = 0.88, p = .38).

### Screening

The screening procedure entailed the implementation of various psychological assessments, including the Theory of Mind Scale (TOM-15, (Desgranges et al., 2012)), the Toronto Alexithymia Scale (TAS-20, (Leising et al., 2009)), the Psychopathic Personality Checklist-Short Version (PCL-SV, (Hart et al., 1995)), the Childhood Trauma Questionnaire-Short Form (CECA.Q,(Bifulco et al., 2005)), the Empathy Calculator (QCAE, (Reniers et al., 2011)). All participants were screened for psychiatric conditions with a trained psychiatrist (MINI, (Lecrubier et al., 1997)). Group differences were explored using two-tailed independent *t*-tests.

### Emotion recognition and emotional rating task

**Fig. 1:**
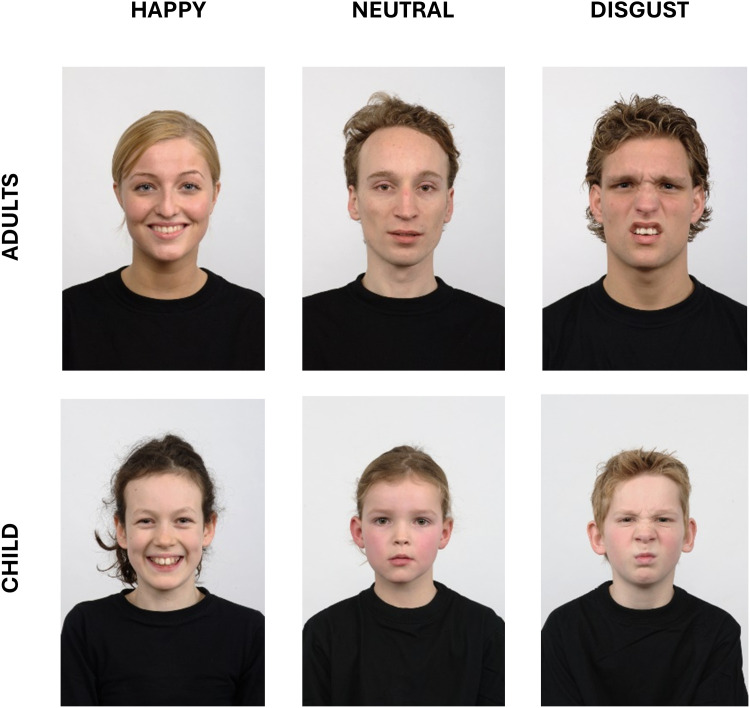
Emotional and age identity were selected from the Radboud Faces Database. The face sample included 16 identities (4 male adults, 4 male children, 4 female adults, and 4 female children). Each faces displaying one of three emotional expressions: happiness, disgust, or neutrality.

Emotion recognition at the behavioral level was assessed using a free viewing task that presented 16 identities from the Radboud Faces Database, each displaying one of 8 emotions (happy, sadness, anger, fear, disgust, surprise, neutrality, or contempt), resulting in a total of 128 stimuli (Fig. 1). The instruction was to select the emotion expressed from 8 possible options without any time limit. Subsequently, the subjects were asked to rate each face on perceived intensity, authenticity, and valence using a 6-point Likert scale.

### Implicit emotion processing

Emotion processing at the neurophysiological level was assessed using an implicit task that presented facial pictures from 16 identities (4 male adults, 4 male children, 4 female adults, and 4 female children (Fig. 1)). Each identity expressed one of 3 emotions: happiness, disgust, or neutrality. This resulted in a total of 48 emotional face stimuli. These stimuli were repeated across 12 presentation blocks, yielding 576 exposures per participant. The stimulus presentation was pseudo-randomized and included a 1-back task to maintain participants’ attention on the emotional faces. Participants were instructed to press the space bar whenever the same face, depicting the same person and emotion, appeared consecutively in the sequence (i.e., “press the space bar when a stimulus is repeated immediately”). Each trial started with the presentation of a fixation cross for a variable duration, ranging from 1250 ms to 1750 ms. After the offset of the fixation cross, an emotional face was displayed for 800 ms. The trial concluded with a blank screen lasting between 2500 ms to 3500 ms.

The EEG signals were recorded using BrainCap electrodes (EasyCAP, GmbH) paired with a 64-channel BrainAmp DC amplifier (Brain Products, GmbH) and BrainVision Recorder software (version 1.25, Brain Products, GmbH). The EEG data were recorded continuously at a sampling rate of 1000 Hz with an electrode impedance kept below 10 kΩ with Abralyt 2000 gel (EasyCAP GmbH). The FCz electrode served as the online reference and the AFz electrode served as the ground. Stimuli were presented using E-Prime 3.0 (Psychology Software Tools USA).

### Behavioral analysis

Statistical analyses were conducted using R software version 4.2.2 and Jamovi version 2.6.44. For the recognition task, participants’ responses were converted into percentages of correct answers and analyzed using a Generalized Linear Model (GLiM). Two control participants were excluded from the analysis due to unavailable data caused by recording issues. The model included Group, Emotion, and Age as fixed effects. The results were reported with χ² (corresponding to the likelihood ratio test statistics), degrees of freedom (df), and p values for each effect. Finally, effect sizes for significant interactions involving Group (patients vs. controls) were reported using Cohen’s d.

Emotional dimension ratings, including valence, intensity, and authenticity, were filtered to include only trials in which the emotion was correctly identified. Then, separate GLiM were conducted for each of the three emotional dimensions. The results were reported with χ², df and p values for each effect. Subsequent post hoc comparisons were performed across conditions using the Bonferroni test method.

### EEG analysis

The analysis of the EEG data was conducted using MNE-Python (Gramfort et al., 2013). A preliminary visual evaluation was conducted to ascertain and interpolate bad sensors, with an average interpolation rate of *M* = 3.7-SD = 2.4 across participants. Subsequently, the continuous EEG data were resampled at a rate of 500 hertz (Hz) and filtered using a 50 Hz notch filter and a 0.1-Hz high-pass filter. This was followed by the rejection of ocular artifacts using Independent Component Analysis (ICA), with an average blink exclusion rate of *M* = 324, SD = 211 across participants. Subsequently, the EEG signal was re-referenced to the average signal and segmented according to the emotional and age conditions (from −2500 ms to +2000 ms relative to the onset of the stimulus). A second step of artifact correction was applied to the segmented signal using the Autoreject library for Python (Jas et al., 2017).

To test for a statistically significant difference in the time domain, we performed a cluster-based permutation analysis using the spatio-temporal cluster test function from MNE-Python. The first analysis used a 3 × 2 factorial design to explore the effects of Emotion Identity (Neutral, Disgust, Happy), Group (SOm vs. Controls), and their interaction (Emotion x Group). A second analysis was performed using a 2 × 2 factorial design to explore the effect of Age Identity (child or adult), Group (SOm vs. Controls), and their interaction (Age x Group). EEG epochs were base line corrected (from −0.250 ms to 0 ms) balanced (*n* = 2716 trials per emotional condition) and averaged per condition across participants. The epochs were organized into contrast matrices corresponding to both factorial designs. Then, we used a two-way repeated measure, cluster-based permutation F-test, on a time window corresponding to the stimulus presentation period (0–800 ms). The significance of each identified cluster was evaluated using a cluster-level threshold set at *p* < .05 and adjusted for multiple comparisons using a cluster-level correction. Post hoc comparisons were then made using bootstrap resampling tests to assess differences in mean amplitudes between groups, separately for each emotion.

## Results

### Profile of sexual offenders

Participant screening was conducted to better characterize the socio-demographic profile of individuals in the SOm group. Notably, 10 individuals in the SOm group reported experiencing unwanted sexual contact during childhood, whereas only one individual in the control group reported a similar experience (Table 2). Furthermore, the SOm group systematically reported more adverse outcomes than the control group across all dimensions related to childhood experiences (Table 3). Regarding the psychiatric interview, two patients from the SOm group self-reported psychiatric condition: one individual with Major Depressive Disorder (MDD) and one with Generalized Anxiety Disorder (GAD). In line with this, although the use of psychotropic medication was an exclusion criterion, we noted three deviations from the protocol base on retrospective examination of medical record: three individuals in the SOm group were receiving no chronic antidepressant treatment (Fluoxetine (20 mg/day), Sertraline (10 mg/day), and Venlafaxine (75mg/day)). However, we cannot exclude the possibility that these medications were prescribed but not actually taken by the participants, as no plasma level verification was included in the study protocol. Seven individuals in the SOm group met the DSM-5 criteria for pedophilic disorder. On the TAS-20 scale, four individuals in the SOm group reached the clinical cutoff for alexithymia (Table 3). The overall TAS-20 scores also differed significantly between groups (t(28) = 3.33, *p* < .01), indicating higher alexithymic traits in the SOm group. In contrast, the overall empathy score (QCAE) and the psychopathy score (PCL-SV) did not differ significantly between groups (*p* > .05).

**Table 2:** Unwanted Sexual Experience or aggression before Age of 17.

Variable		SOm (n = 15)			Control (n = 15)	
		n	%		n	%
Unwanted sexual Experience during childhood/adolescence						
	Yes	10	67		1	7
	No	2	13		14	93
	Uncertain	3	20		0	0
Sexual intercourse						
	Yes	9	60		0	0
	No	4	27		15	100
	Uncertain	1	7		0	0
With an adult or authority figure						
	Yes	5	33		1	7
	No	9	60		14	93
	Uncertain	1	7		0	0

**Table 3:** Raw screening scores for the SOm and control groups.

	SOm (n = 15)		Control (n = 15)	
	Mean	SD	Mean	SD
CECA Overall Dimension				
Antipathy Overall Score (/160)	18,3	(9,3)	11,9	(5,0)
Neglect Overall Score (/160)	16,4	(6,2)	10,5	(2,7)
Physical Abuse Overall Score (/8)	1,3	(1,5)	0,2	(0,6)
Presence of Non-consensual Sexual Exp (/3)	1,9	(0,9)	0,1	(0,4)
Prevalence of Psychological Abuse (/34)	7,5	(7,2)	1,9	(2,9)
Frequency of Psychological Abuse (/102)	10,9	(10,5)	2,7	(3,6)
QCAE Total Score (/124)				
	79,9	(11,7)	81,3	(14,9)
PCL-SV factors and total scores*				
Score Factor-1 (/12)	1,85	(3,31)	0,82	(1,25)
Score Factor-2 (/12)	1,69	(2,9)	1,09	(1,38)
Overall Score (/24)	3,54	(5,87)	1,91	(2,59)
TAS-20 Total Score (/100)				
	52,5	(13,7)	39,3	(6,9)
	n	%	n	%
Cut-off TAS				
Alexithymia (≥61*)	4	26,7	0	0
Possible alexithymia (52–60)	4	26,7	0	0
Non-alexithymia (≤51)	7	46,7	15	100

⁎ One individual in the SOm group reaches the cut-off > 18.

### Behavioral results: emotion recognition task

**Fig. 2:**
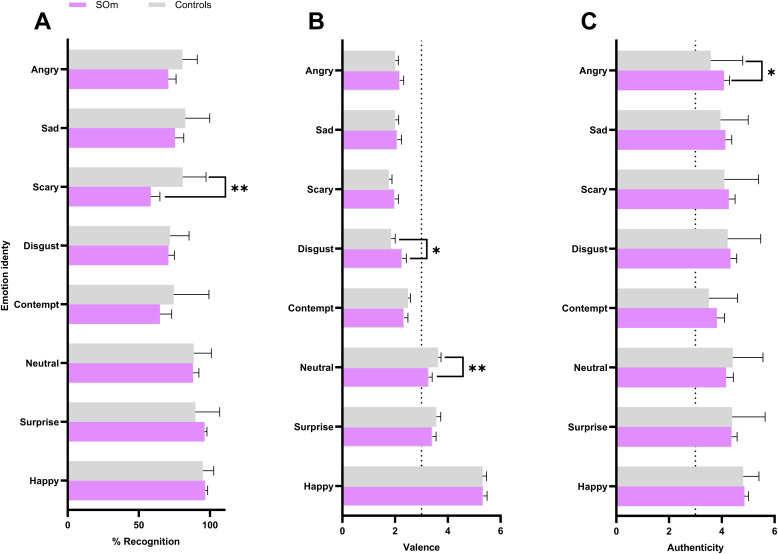
Mean emotion recognition accuracy and rating dimension by emotion identity × group (SOm: cyan; Controls: grey). Error bars represent ±1 standard error of the mean (SEM). A) A significant Emotion × Group interaction was observed in the GLiM analysis for %Recognition. The post hoc comparisons revealing a significant difference for the "Fear" emotion (*pBonf <0.05; **pBonf < 0.01). B) A significant Emotion × Group interaction was observed with post-hoc difference for the "disgust" and “neutral” for Valence rating. C) A significant Emotion × Group interaction was observed with post-hoc difference for the "angry" emotion in authenticity rating. Note : No significant difference was found for Authenticity rating. Raw data for % Recognition, Valence, Intensity and Authenticity were reported in supplementary material.

The GLiM model revealed significant main effects of Emotion (χ²(7) = 79.96, *p <* .001), Group (χ²(1) = 8.16, *p <* .01) and Emotion × Group interaction (χ²(7) = 18.84, *p <* .01, Cohen’s *d* = 0.67) on recognition. These results indicate that SOm performed worse overall in recognizing emotions compared to the control group and that recognition accuracy differed between groups depending on the specific emotion. Post hoc analyses showed that this interaction was primarily driven by the "Fear" emotion (Z(406) = 4.43, *pBonf* < 0.001, Fig. 2A), with SOm recognizing only 58.34 % of the stimuli (SD = 24.54 %), compared to 80.76 % (SD = 16.43 %) for controls. Neither the main effects of Age (χ²(1) = 0.005, *p =* .76), Emotion × Age (χ²(1) = 9.928, *p =* .19) nor the three-way interaction between Emotion × Group × Age (χ²(7) = 2.56, *p =* .95) reached statistical significance.

### Behavioral results: emotional rating task

The GLim model for valence rating reported main effect of emotion (χ²(7) = 3878.74, *p* < .001), Emotion × Age (χ²(7) = 18.78, *p* < .01) and Emotion × Group interaction (χ²(7) = 34.35, *p* < .001, Cohen’s *d* = 0.47, Fig. 2B). Post-hoc analyses revealed that SOm rated neutral (*M* = 3.27, SD = 0.56) emotional identities as more negatively valanced (Z(2607) = 3.98, *pBon*f < 0.01, Fig. 2B), but rated disgust emotional identities as more positively valanced (*M* = 2.26, SD = 0.62) than controls (Z(2607) = 3.61, *pBonf* < 0.05, Fig. 2B; neutral: *M* = 3.62, SD = 0.40; disgust: *M* = 1.85, SD = 0.51). Neither the main effects of Age (χ²(1) = 0.006, *p* = .94), Group × Age (χ²(1) = 0.004, *p* = .95) nor the three-way interaction between Emotion × Group × Age (χ²(7) = 0.96, *p* = .99) reached statistical significance.

Regarding the intensity of the model's findings, a main effect of Emotion was reported (χ²(7) = 686.48, *p* < .001). The analysis revealed no statistically significant main effect of Group (χ²(1) = 0.31, *p* = .57) or Age (χ²(1) = 3.67, *p* = .06, ). Additionally, the interaction between Group × Age (χ²(1) = 0.28, *p* = .59) and Group × Emotion (χ²(7) = 8.68, *p* = .27) was not significant. The three-way interaction (Group × Age × Emotion; χ²(7) = 2.99, *p = .*88) was also not significant.

Finally, the model for authenticity ratings also showed a main effect of Emotion (χ²(7) = 133.92, *p* < .001), as well as significant effects of Group (χ²(1) = 4.65, *p* = .031) and Age (χ²(1) = 6.84, *p* < .01). Significant interactions were found between Group × Emotion (χ²(7) = 26.08, *p* < .001, Cohen’s *d* = 0.53, Fig. 2C) with control rating less authenticity for angry faces (*M* = 3.58, SD = 1.22) than SOm (Z(2607) = −4.40, *pBonf* < 0.05, Fig. 2C; *M* = 4.08, SD = 0.8). The Age × Emotion interaction was also significant (χ²(7) = 28.60, *p* < .001) but no significant post-hoc comparison was found. Finally, the three-way interaction (Group × Age × Emotion recognition) was not significant (χ²(7) = 3.77, *p* = .88).

### EEG results: implicit emotion processing

**Fig. 3:**
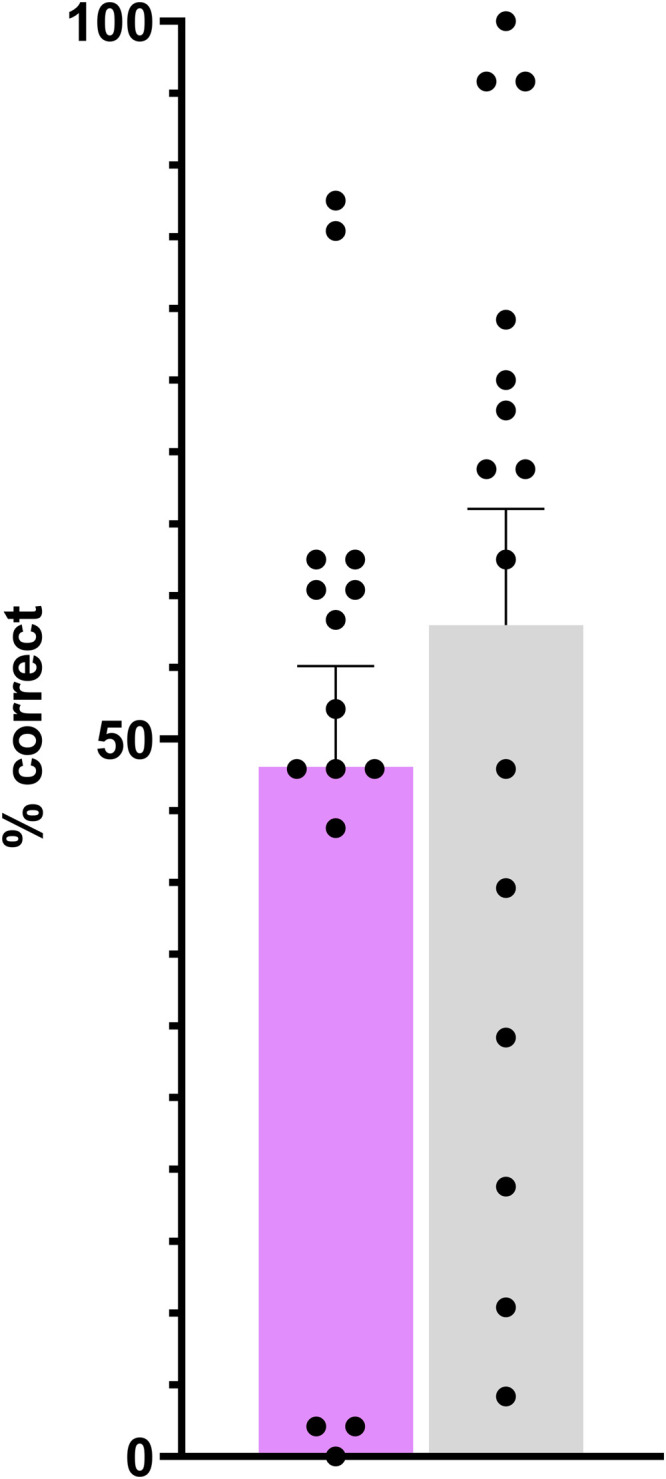
Percentage of correct responses in the emotion 1-back task by group. No difference was observed between groups (p < 0.05); Purple = SOm group, Grey = control group. Each dot corresponds to an individual participant (n = 15 per group). Bar error = SEM.

The percentage of correct 1-back responses did not significantly differ between groups. The SOm group had a mean accuracy of *M* = 48.1 %, SD = 27.18 %, while the control group showed *M* = 57.9 %, SD = 31.4 %. This difference was not statistically significant, t(28) = 0.921, *p* = .59 (Fig. 3). The low performance observed in three SOM participants and one healthy individual could reflect either a misunderstanding of the task or a strong interference effect from the primary free-viewing paradigm.

**Fig. 4:**
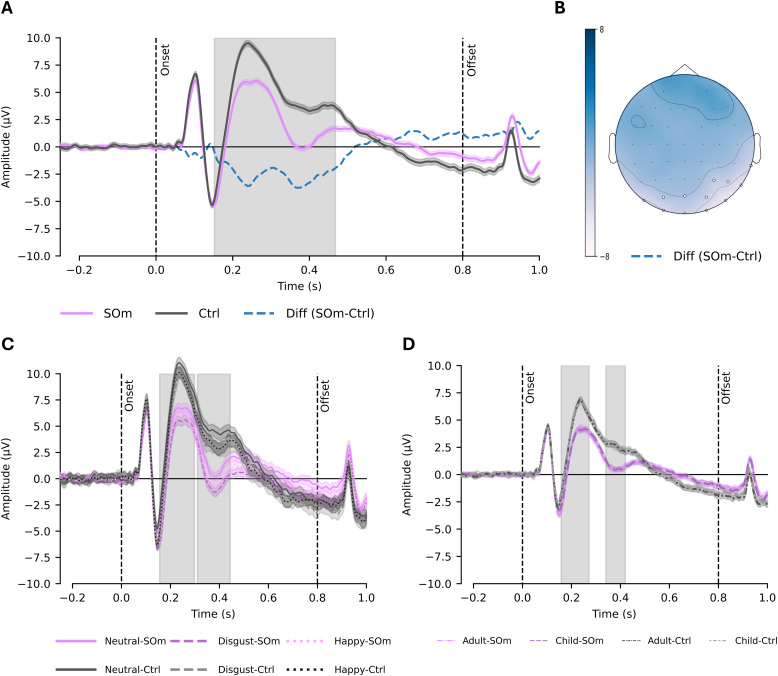
Grey zones - significant time windows (**p < .001). A) Cluster for the main effect of group: the blue line represents the difference in evoked amplitudes between SOm (SOm – purple line) and controls (Ctrl – grey line) following stimulus onset (p < .01). B) The topomap illustrates the mean difference between the SOm and control groups within the significant time window (152–458 ms). C) Emotion × Group interaction: two significant clusters emerged over occipito-parietal electrodes. The first cluster, within the P2 latency window (156–298 ms), included sensors O2, P8, PO4, P6, PO8, O1, and PO7. Post hoc comparisons revealed significantly reduced amplitudes in the SOm group compared to controls for neutral and disgust expressions (p < .01). The second cluster, within the LPP time range (334–444 ms) and involving the same sensors, showed a significant reduction in amplitude for neutral expressions in the SOm group relative to controls (p < .01). D) Age × Group interaction: the first significant cluster (158–272 ms) over posterior electrodes (O2, P8, PO4, P6, PO8) corresponds to the P2 latency window. Post hoc analyses indicated significantly lower amplitudes in the SO group for both child and adult face identities (p < .05). A second cluster (340–420 ms; same sensors) within the LPP time window revealed reduced amplitudes for child identities in the SO group compared to controls (p < .05), with no significant difference for adult faces. Diff=Difference, SOm = Sexual offender against minor; Ctlr =Controls.

A cluster-based spatiotemporal analysis revealed a significant main effect of group over a time window in occipito-parietal electrodes (Fig. 4B) from 152 ms to 468 ms (*p* < .01). This indicates reduced amplitude in the SOm group compared to controls following stimulus onset (M-diff = −1.7 µV, 95 %CI [−2.79, −0.59], *p* < .01), Fig. 4A).

Additionally, a significant emotion × group interaction was identified, with two clusters emerging over occipito-parietal electrodes between 156 ms and 444 ms. The first cluster corresponded to the typical P2 latency window (156ms–298 ms; *p* < .001), Fig. 4C) and showed a significant amplitude reduction in the SOm group for the neutral and disgust identities compared to the control group (M-diff = −2.43 µV, 95 %CI [−4.60, −0.28], *p* < .05 and M-diff = −2.29 µV, 95 %CI [−4.34, −0.21], *p* < .01, respectively). Interestingly, these 2 emotions correspond to the significant differences observed in the valence ratings. The second cluster (334ms–444 ms; *p* < .001, Fig. 4C), reflecting a later negative deflection consistent with the LPP time window show significant amplitude reduction for neutral identity in SOm compared to controls (M-diff = −2.46 µV, 95 %CI [−4.83, −0.01]). No other post hoc comparisons reach statistical significance (*p* > .05). Finally, the main effect of Emotion did not yield any statistically significant cluster *(p* > .01).

Regarding Age identity (Adult vs. Child) and group (SOm vs. Controls) a significant main effect of group was observed in a cluster spanning from 122 ms to 488 ms post-stimulus onset (*p* < .01). However, post hoc comparisons did not reach statistical significance (M-diff = −1.41 µV, 95 % CI [−2.82, 0.03], *p* > .05). The age × group interaction revealed two significant clusters in the P2 latency range (158–272 ms; *p* < .001) and in the LPP range (340–420 ms; *p* < .001). Post hoc analyses of the early cluster showed significantly reduced amplitudes in the SO group compared to the control group for both child (mean diff = −2.28 µV, 95 % CI [−4.44, −0.10], *p* < .05) and adult (mean difference = −2.43 µV, 95 % CI [−4.51, −0.28], *p* < .05) identities. In the later cluster, a significant reduction in amplitude was also observed in the SOm group for child identities (mean difference = −2.45 µV, 95 % CI [−4.70, −0.06], *p* < .05, - Fig. 4D), though the difference for adult faces did not reach significance (M-diff = −2.30 µV, 95 % CI [−4.68, 0.20], *p* > .05).

## Discussion

The present study was specifically designed to investigate facial emotion perception and recognition key components of social cognition at both behavioral and neurophysiological levels in individuals convicted of sexual offenses against minors, compared with controls. As expected, group differences emerged at both the behavioral level and in early and late ERP components, depending on the emotional valence and the age of the faces presented. Notably, the current EEG results provide new insights into the abnormal processing of emotional stimuli in SOM individuals. Interestingly, early attentional components such as the P1 and N1/N170 were largely preserved, showing no significant group differences. In contrast, the absence or reduction of later components associated with attentional process and emotional recognition constitutes a strong marker of atypical emotional face processing in this population.

### Discussion of behavioral results

The behavioral results regarding facial emotion recognition show a general tendency towards misrecognition of emotion, particularly of fearful facial expressions in the SOm group compared to the control group. These results are in line with previous finding (Gillespie et al., 2015). Additionally, the observation that individuals from the SOm group rated disgust facial expressions as positive compared to control group is also consistent with previous findings (Francis & Wolfe, 2008). However, the hypothesis that face age influences emotional recognition and perception was not supported by the results. One possible explanation for the absence of differences is the age of the children depicted in the images. In the study by Speer et collaborators (2022), the children in the pictures ranged from 2 to 8 years old, whereas the Radboud database features children closer to 12 years old.

### Discussion of EEG results

The reduced amplitude of the P2 component in the SOm group following stimulus onset aligns with previous findings (Knott et al., 2016). The P2 component, which is typically distributed over the temporal and parieto-occipital locations, is considered an EEG marker of emotional salience processing in response to emotional facial expressions (Dennis & Chen, 2008). The P2 component has been widely associated with emotion processing and various psychopathological conditions. For example, individuals with psychopathic traits have shown reduced P2 amplitudes in response to emotional stimuli (Clark et al., 2019). Similarly, among individuals with psychopathy or externalizing disorders linked to aggressive behavior, reduced P2 amplitudes were observed when processing emotional stimuli (Anderson & Stanford, 2012; Willner et al., 2020). These observations of blunted P2 amplitudes in these populations can be interpreted as a reduced allocation of attentional resources toward emotional stimuli. Interestingly, previous study (Willner et al., 2020) suggested that the enhanced P2 amplitude for neutral faces may reflect a hostile attribution bias, where neutral expressions are perceived as ambiguous and potentially threatening. In the current study, a similar trend was observed in the behavioral results, with the SOm group providing more negative ratings for neutral faces compared to the control group. Additionally, enhanced P2 amplitudes were observed in response to neutral faces in the SOm group; however, this effect did not reach statistical significance. Interestingly, no cluster emerged during the early attentional and perceptual processing. The N170 and P1 component appears to be preserved in the SOm group, indicating efficient categorization and face processing (Jiang et al., 2009; Luo et al., 2010). This result differs from previous observations of enhanced P1 during the processing of child faces in juvenile sexual offenders with a prepubescent preference (Speer et al., 2022). The absence of significant differences in the current study may be influenced by the limited number of individuals with paraphilic disorders in the sample. Indeed, compared to the study by Speer and colleagues (2022), only seven individuals in our sample were diagnosed with pedophilia.

Regarding later components, numerous EEG studies have demonstrated that the LPP is typically larger in response to emotional and arousing stimuli compared to neutral ones (Hajcak et al., 2010). Nevertheless, the functional significance of the LPP component should not be reduced solely to emotional processing. It is suggested by several studies that the LPP also reflects the motivational relevance of stimulus. That is to say, the extent to which a stimulus activates appetitive or aversive motivational systems (Fields, 2023). To date, only 1 study has investigated the LPP component during the presentation of erotic stimuli in SOm population and reported no significant group differences compared to controls (Knott et al., 2016). However, in the offender populations, hyporeactivity reflected by reduced LPP amplitudes in response to emotional stimuli is well documented. For instance, a lack of LPP modulation in response to emotionally valanced stimuli has been reported in juvenile and psychopathic offender populations (Pincham et al., 2015; Sadeh & Verona, 2012). Regarding anatomical structure of the brain previous works have reported the implication of the occipital cortex, the amygdala, the temporal areas, the prefrontal cortex (PFC) and the orbitofrontal cortex (OFC) in the possible source for generation of LPP (Liu et al., 2012). Interestingly, abnormalities in these brain regions have also been associated with sexual offending behavior and pedophilia (Ara-García et al., 2025; Klöckner et al., 2021; Poeppl et al., 2015; Schiffer et al., 2008, 2017; Vallet et al., 2019)

In the current study, the observation of an LPP component is congruent with previous observation during a passive viewing paradigm using emotional faces (Fields, 2023). Classically, the LPP component emerges around 400 ms after stimulus onset and can last for several hundred milliseconds or more. In the current study, the stimulus presentation time was limited to 800 ms, which may partly explain the absence of a main effect of emotion on LPP modulation. Nevertheless, the current study is the first to demonstrate abnormalities in emotional stimulus processing in the SOm population, characterized by a marked reduction or absence of the LPP component compared to the control group.

### Limits regarding the clinical characteristics of the included population

Despite the absence of a universally accepted typological classification of sexual offenders, a widely used and legally recognized distinction is based on the age of the victim, allowing for the differentiation between offenders targeting adults and those targeting minors (Wojcik & Fisher, 2019). Among sexual offenders who have committed sexual assault against minors, a subset can be classified as pedophiles. Contrary to sexual offending against minors (SOm), pedophilia is not a legal or criminal classification, but rather a psychiatric disorder referenced in the DSM (American Psychological Association, 2017). The disorder or paraphilia is characterized by persistent and recurrent sexually arousing fantasies or urges (lasting >6 months) involving prepubescent individuals under the age of 13. However, pedophilia is not a prerequisite for committing sexual assaults against minors. Nevertheless, it remains a significant risk factor, as it is strongly associated with a higher prevalence of child sexual abuse. Research indicates that between 22 % and 43 % of individuals with pedophilic interests admit to having engaged in sexual contact with a minor under the age of 13 (Seto, 2012). These self-reported data are corroborated by findings indicating that approximately 50 % of convicted minor sexual offenders report a pedophilic sexual preference (Gerwinn et al., 2018; Whitaker et al., 2008). In the current study, 7 SOm could be classified as pedophilic according to DSM-5 criteria; however, the limited size of this subsample did not allow for further investigation.

The emotional recognition deficits identified in the SOm group may also be confounded by other comorbidities commonly observed within the broader typology of violent and sexual offending. Psychiatric conditions, particularly MDD and anxiety-related disorders such as post-traumatic stress disorder (PTSD), are commonly reported and have a high prevalence among this population. In convicted or incarcerated populations, the prevalence of psychiatric disorders ranges from 23 % to 51 % (Baranyi et al., 2022; Majekodunmi et al., 2017). When studying emotional processing, MDD is a potential confounding factor. A recent meta-analysis (Krause et al., 2021) found that individuals with MDD, particularly those with severe symptoms, exhibited lower accuracy in recognizing facial expressions than those with moderate symptoms. However, among the SOm and control participants included in the current study, only 1 individual was diagnosed with MDD. Therefore, while depressive symptoms can influence emotional recognition abilities, the observed deficits in the SOm group are unlikely to be fully attributed to depression as a confounding variable.

Generalized Anxiety Disorder (GAD) is another psychiatric comorbidity that may interfere with emotion recognition abilities. GAD appears to have a specific effect on emotion recognition, varying according to the typology of violent and sexual population studied and the emotion being expressed. Meta-analyses have consistently reported significant impairments in emotion recognition among individuals with GAD (Baez et al., 2023; Demenescu et al., 2010). Interestingly, the nature of these impairments may be emotion specific. For instance, individuals with anxiety disorders were significantly better at recognizing fearful facial expressions than healthy controls, suggesting a heightened sensitivity to threat-related cues (Surcinelli et al., 2006). In sexual offenders against adults and minors, higher levels of anxiety have been reported compared to non-sexual offenders and control groups (Kanters et al., 2016). Personality disorders, particularly psychopathy, are also commonly reported as comorbidities in SOm (Turner et al., 2014), with prevalence estimates ranging from 10 % to 15 % in this population. Nonetheless, existing literature provides robust evidence linking psychopathy to deficits in emotion recognition, especially for negative facial expressions such as fear, disgust, and sadness (Blair, 2018; De Brito et al., 2021).

### General discussion

The main goal of the current study was to explore and analyze electrophysiological processing in the SOm population, with the aim of identifying abnormalities in brain activity associated with emotional processing. In some cases, these electrophysiological signal specifics may be extrapolated cautiously as potential biomarkers and associated with the population of interest (i.e. individuals who have committed sexual offences against children). However, the term 'biomarker', emerging from studies conducted on heterogeneous samples, should be used with caution (Ramião et al., 2023). For example, literature identifies several subcategories within the population sexually attracted by children, which correspond to different stages of pubertal development (Blanchard et al., 2001; Stephens et al., 2017) and specific brain activation during the processing of sexual arousal (Mohnke et al., 2014).

In conclusion, these results highlight the complexity of studying individuals who have offended children due to the diversity of their phenotypes and comorbidities (Virolle et al., 2024). This challenge is further exacerbated by their frequent incarceration, which restricts researchers' access to this population. The current results showing deficits in emotional neural processing and recognition should be interpreted with caution, and further studies are needed to establish reliable biomarkers for the prediction of aggressive behaviors.

## Declaration of competing interest

The authors declare the following financial interests/personal relationships which may be considered as potential competing interests:

William Vallet reports financial support was provided by Centre hospitalier le Vinatier. If there are other authors, they declare that they have no known competing financial interests or personal relationships that could have appeared to influence the work reported in this paper.

## Appendix Supplementary materials

Image, application 1
