# Supplementary material for: A combined behavioral and EEG investigation of facial emotion recognition in sexual offenders against minors
Source: Int J Clin Health Psychol. 2025 Oct 15;25(4):100641. doi: 10.1016/j.ijchp.2025.100641 (PMC12550296; doi:10.1016/j.ijchp.2025.100641)
Supplement: Supplementary file 1 [file mmc1.docx]

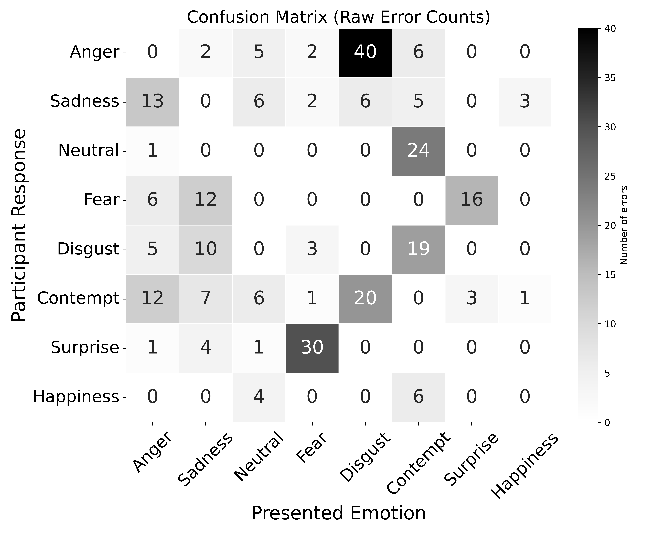

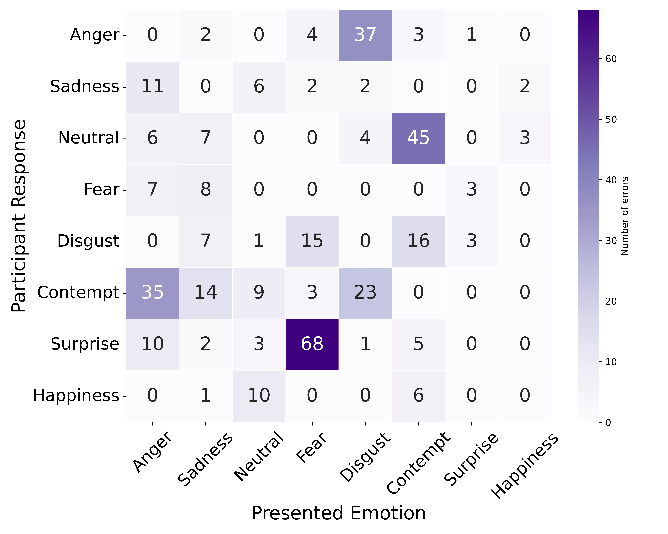


**Figure-S1**: The shows the number of incorrect responses by emotion category in Som (left) and controls (right). The rows represent the participants' selected responses, and the columns indicate the actual emotions presented. Greater confusion between specific emotion categories is indicated by higher values of the diagonal.

**Table S1** - Raw data for emotional recognition task per emotion :

| **Emotion Identity** | **SOm (n=15)** | | **Controls (n=15)** | |
| --- | --- | --- | --- | --- |
|  | Mean% | SD% | Mean% | SD% |
| ***Happy*** | 96.66 | 6.19 | 95.09 | 6.97 |
| ***Surprise*** | 96.16 | 6.38 | 89.74 | 17.63 |
| ***Neutral*** | 88.08 | 15.65 | 88.59 | 11.45 |
| ***Contempt*** | 64.77 | 24.83 | 74.47 | 25.04 |
| ***Disgust*** | 70.87 | 15.26 | 71.827 | 13.60 |
| ***Fear***** | 58.34 | 24.54 | 80.76 | 16.43 |
| ***Sad*** | 75.39 | 11.78 | 82.69 | 17.15 |
| ***Angry*** | 70.75 | 21.38 | 80.57 | 11.46 |

**Table S2.** Raw data for emotional valance rating :

| **Emotion Identity** | **SOm (n=15)** | | **Controls (n=15)** | |
| --- | --- | --- | --- | --- |
|  | Mean% | SD% | Mean% | SD% |
| ***Happy*** | 5.34 | 0.56 | 5.32 | 0.51 |
| ***Surprise*** | 3.41 | 0.52 | 3.56 | 0.60 |
| ***Neutral***** | 3.27 | 0.51 | 3.63 | 0.39 |
| ***Contempt*** | 2.33 | 0.56 | 2.49 | 0.31 |
| ***Disgust**** | 2.26 | 0.61 | 1.85 | 0.51 |
| ***Fear*** | 1.98 | 0.52 | 1.76 | 0.42 |
| ***Sad*** | 2.08 | 0.60 | 2.00 | 0.47 |
| ***Angry*** | 4.08 | 0.82 | 3.57 | 1.21 |

**Table S3.** Raw data for emotional authenticity rating :

| **Emotion Identity** | **SOm (n=15)** | | **Controls (n=15)** | |
| --- | --- | --- | --- | --- |
|  | Mean% | SD% | Mean% | SD% |
| ***Happy*** | 4.86 | 0.56 | 4.81 | 0.59 |
| ***Surprise*** | 4.36 | 0.82 | 4.39 | 1.24 |
| ***Neutral*** | 4.16 | 1.07 | 4.41 | 1.14 |
| ***Contempt*** | 3.81 | 1.08 | 3.50 | 1.08 |
| ***Disgust*** | 4.32 | 0.91 | 4.22 | 1.25 |
| ***Fear*** | 4.26 | 0.88 | 4.09 | 1.29 |
| ***Sad*** | 4.13 | 0.89 | 3.95 | 1.04 |
| ***Angry**** | 4.08 | 0.82 | 3.57 | 1.21 |

**Table S4*.*** Raw data for emotional intensity rating :

| **Emotion Identity** | **SOm (n=15)** | | **Controls (n=15)** | |
| --- | --- | --- | --- | --- |
|  | Mean% | SD% | Mean% | SD% |
| ***Happy*** | 4.68 | 0.60 | 4.43 | 0.86 |
| ***Surprise*** | 4.37 | 0.72 | 4.63 | 0.64 |
| ***Neutral*** | 2.99 | 0.92 | 2.82 | 1.23 |
| ***Contempt*** | 3.10 | 0.65 | 3.08 | 1.25 |
| ***Disgust*** | 4.58 | 0.79 | 4.68 | 0.78 |
| ***Fear*** | 4.50 | 0.62 | 4.72 | 0.61 |
| ***Sad*** | 3.94 | 0.65 | 3.77 | 0.89 |
| ***Angry*** | 3.99 | 0.90 | 4.00 | 0.85 |

**Table S5.** Generalized linear model: Emotion recognition task

| ***Model summary*** |  | AIC | X^2^ | p-value |
| --- | --- | --- | --- | --- |
|  |  | 3900.89 | 66324 | p < .001*** |
| ***ANOVA summary*** |  | df | χ² | p-values |
| *Predictor* |  |  |  |  |
| *Emotion* | | 7 | 114.28 | p < .001*** |
| *Group* | | 1 | 8.16 | p < .01** |
| *Age* | | 1 | 0.01 | p > .05 |
| *Emotion ✻ Group* | | 7 | 21.09 | p < .01** |
| *Emotion ✻ Age* | | 7 | 9.93 | p > .05 |
| *Group ✻ Age* | | 1 | 0.032 | p > .05 |
| *Emotion ✻ Group ✻ Age* | | 7 | 2.56 | p > .05 |

**Table S6.** Generalized linear model: Valance rating task

| ***Model summary*** |  | AIC | X^2^ | p-value |
| --- | --- | --- | --- | --- |
|  |  | 6920.56 | 362 | p < .001*** |
| ***ANOVA summary*** |  | df | χ² | p-values |
| *Predictor* |  |  |  |  |
| *Emotion* | | 7 | 3878.53 | p < .001*** |
| *Group* | | 1 | 0.004 | p > .05 |
| *Age* | | 1 | 0.006 | p > .05 |
| *Emotion ✻ Group* | | 7 | 34.35 | p < .01** |
| *Emotion ✻ Age* | | 7 | 18.75 | p < .01** |
| *Group ✻ Age* | | 1 | 0.047 | p > .05 |
| *Emotion ✻ Group ✻ Age* | | 7 | 0.96 | p > .05 |

**Table S7.** Generalized linear model: Authenticity rating task

| ***Model summary*** |  | AIC | X^2^ | p-value |
| --- | --- | --- | --- | --- |
|  |  | 9889.10 | 362 | p < .001*** |
| ***ANOVA summary*** |  | df | χ² | p-values |
| *Predictor* |  |  |  |  |
| *Emotion* | | 7 | 133.92 | p < .001*** |
| *Group* | | 1 | 4.65 | p < .05* |
| *Age* | | 1 | 6.83 | p < .01** |
| *Emotion ✻ Group* | | 7 | 26.07 | p < .001*** |
| *Emotion ✻ Age* | | 7 | 28.59 | p < .001*** |
| *Group ✻ Age* | | 1 | 0.31 | p > .05 |
| *Emotion ✻ Group ✻ Age* | | 7 | 3.77 | p > .05 |

**Table S7*.*** Generalized linear model: Intensity rating task

| ***Model summary*** |  | AIC | X^2^ | p-value |
| --- | --- | --- | --- | --- |
|  |  | 9106.10 | 76.6 | p < .001*** |
| ***ANOVA summary*** |  | df | χ² | p-values |
| *Predictor* |  |  |  |  |
| *Emotion* | | 7 | 686.47 | p < .001*** |
| *Group* | | 1 | 0.310 | p > .05 |
| *Age* | | 1 | 3.67 | p > .05 |
| *Emotion ✻ Group* | | 7 | 8.67 | p > .05 |
| *Emotion ✻ Age* | | 7 | 28.11 | p < .001*** |
| *Group ✻ Age* | | 1 | 0.28 | p > .05 |
| *Emotion ✻ Group ✻ Age* | | 7 | 2.98 | p > .05 |
